# COVID-19 Positive in Nasopharyngeal Swab but Negative in Peritoneal Fluid: Case Report of Perforated Appendicitis

**DOI:** 10.7759/cureus.9412

**Published:** 2020-07-27

**Authors:** Lahari Vudayagiri, John Gusz

**Affiliations:** 1 General Surgery, Western Reserve Hospital, Cuyahoga Falls, USA; 2 General Surgery, University Hospital Portage Medical Center, Ravenna, USA

**Keywords:** covid-19, peritoneal fluid

## Abstract

COVID-19 has drastically changed hospital systems from a microcosmic to macrocosmic level, specifically for surgical practices worldwide. COVID-19 surgical guidelines are continuing to evolve as we deepen our understanding of the virus. A particular point of interest is the possibility of aerosolization of COVID-19 during laparoscopic procedures. There is much uncertainty of the pathogenicity of COVID-19 and insufficient data on the presence and extent of viral load in different body fluids, specifically in peritoneal fluid.

We present a case of a 27-year-old male who was diagnosed with acute appendicitis and found to be COVID-19 positive postoperatively. Intraoperative peritoneal fluid sampling was obtained and tested for COVID-19 through real-time reverse transcription polymerase chain reaction (RT-PCR) targeting N1 and N2 proteins. COVID-19 was not detected in RT-PCR test in the peritoneal fluid collected; however, it was detected in the nasopharyngeal RT-PCR. The patient had prolonged stay in the hospital secondary to COVID-19 symptoms.

Currently, there is very limited and inconclusive evidence on the presence of COVID-19 in peritoneal fluid. We present the first paper discussing perforated bowel, in which COVID-19 is not detected in peritoneal fluid. This case report provides more insight regarding shaping guidelines for surgeries in patients with COVID-19.

## Introduction

COVID-19 is an overwhelming presence throughout all hospital systems as hospitals become the primary center for both treatment and risk of transmission of this disease. Surgical subspecialties have specifically been affected by this pandemic. Operating rooms are high-risk areas for the transmission of air-borne infections given the presence of multiple medical personnel in close proximity, high levels of aerosolized virus given the predominance of airway management under anesthesia, lack of personal protective equipment (PPE), and limited availability of negative pressure operating rooms. Hence, the surgical guidelines continue to evolve at the local, national, and international levels for the protection of both patients and staff against COVID-19 [1,2].

American College of Surgeons (ACS) and Society of American Gastrointestinal and Endoscopic Surgeons (SAGES) guidelines note that aerosol generating procedures such as electrocautery, endoscopy, and in particular, laparoscopy cause unavoidable increased risk to healthcare workers [1,2]. ACS guidelines even suggest that laparoscopy should only be used in specific cases when the clinical benefit to the patient exceeds the risk of exposure to the healthcare staff [1]. SAGES guidelines regarding laparoscopy state that incisions for ports be “as small as possible” for minimal leakage of gas around ports, minimum CO_2_ insufflation, and evacuation of all of the pneumoperitoneum be through a closed filtration system prior to termination of the laparoscopic procedure [2]. However, there is currently insufficient data to prove aerosolization of COVID-19 via laparoscopy. Here we present a case of laparoscopic appendectomy performed on patient with an unknown COVID status preoperatively, who was positive for COVID-19 postoperatively via nasopharyngeal reverse transcriptase polymerase chain reaction (RT-PCR), but had no detection of COVID-19 in peritoneal fluid by RT-PCR.

## Case presentation

**Figure 1:**
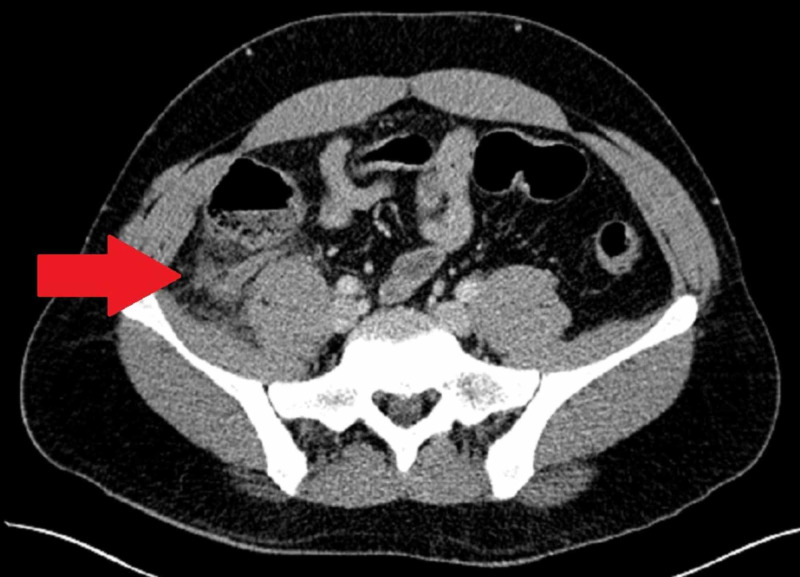
Perforated appendicitis on abdominal CT scan

A 27-year-old male patient with no significant past medical or surgical history presented to a community hospital with acute onset of right lower quadrant abdominal pain radiating into the right flank for one day. On admission, he denied fever, shortness of breath, and cough. Physical exam revealed tenderness at McBurney’s point without peritoneal signs. His significant laboratory findings on admission included leukocytosis of 13.8 x 10^3^/mm^3^ with normal lymphocyte count. A CT scan of the abdomen and pelvis with IV contrast confirmed acute appendicitis. The patient had a dilated 12 mm appendix and extensive periappendiceal soft tissue stranding and a small amount of free fluid in the right lower quadrant, raising concern for perforation (Figure 1). A limited evaluation of his lungs as seen in the CT abdomen and pelvis showed no acute abnormalities. As he was saturating greater than 95% on room air with no apparent COVID-19 symptoms, chest x-ray and COVID-19 testing were not performed preoperatively.

On the day of admission, the patient underwent laparoscopic appendectomy. The patient was intubated with staff present in N95 masks. He underwent a standard three-port laparoscopic appendectomy with CO_2_ insufflation. Upon entrance into the abdomen, the patient had turbid fluid in the pelvis which was suctioned and sent for culture. The appendix itself appeared gangrenous but was successfully removed intact using a laparoscopic endocatch bag. The pneumoperitoneum was evacuated without the use of a closed filter, and the patient was extubated and tolerated the procedure well.

Postoperatively, the patient was noted to be intermittently febrile (max temperatures of 38.1°C) and COVID-19 nasopharyngeal swab was performed. Intraoperative peritoneal fluid was also sent for COVID-19 testing. Both peritoneal fluid and nasopharyngeal fluid were analyzed via real-time RT-PCR using the modified CDC assay. Gram stain and aerobic and anaerobic cultures of the peritoneal fluid were positive for Streptococcus anginosus. Nasopharyngeal swab was positive for COVID-19, and the patient was subsequently isolated with contact precautions. Chest x-ray was performed after the patient was found to be COVID-19 positive, which showed no acute abnormalities. Postoperatively his leukocytosis resolved. He was noted to have elevated C-reactive protein (CRP) at 13 mg/L and normal lactate dehydrogenase. He was placed on ampicillin-sulbactam for empiric antibiotic coverage. Despite progressing through his hospital stay, he continued to be intermittently febrile. He was discharged on postoperative day 4 when he was afebrile and asymptomatic to quarantine in his home for two weeks.

Results

COVID-19 was detected in the nasopharyngeal swab RT-PCR and was not detected in the peritoneal fluid RT-PCR, which were both performed via the real-time RT-PCR using the modified CDC assay that detects both N1 and N2 nucleoprotein targets.

## Discussion

Currently, there is limited information regarding aerosolization of COVID-19 intraoperatively with rapidly evolving protocols and guidelines. After this case (and in following national guidelines), our hospital has several protocols for both elective and urgent procedures. Currently, all patients for elective and emergent procedures undergo COVID-19 screening preoperatively and PPE is required around any patients with detected COVID-19. Anyone present in the room for endoscopic procedures and all anesthesia staff are required to wear PPE for all patients. Furthermore, PPE is recommended, but not enforced, for all operative procedures. The current guidelines are so broadly based secondary to the amount of uncertainty in pathogenesis of COVID-19. Although COVID-19 is primarily transmitted through respiratory droplets, there have been several publications discussing the detection of COVID-19 in sputum, feces, blood, and even cerebrospinal fluid [3-5]. However, there is limited information guiding protocols regarding aerosolization of COVID-19. 

There have been limited publications to date discussing COVID-19 and detection in peritoneal fluid. Multiple studies have sampled peritoneal fluid; however, only one publication detects COVID-19 within peritoneal fluid [6]. This publication by Coccolini et al. noted a higher viral load in peritoneal fluid compared to the upper respiratory tract in a COVID-19 positive patient presenting with a small bowel obstruction [6]. Another case report of a patient with non-perforated appendicitis analyzed peritoneal fluid in which COVID-19 was not detected [7]. It is important to note the methods of detection of COVID-19 in these studies. Our study is the first to evaluate peritoneal fluid in a perforated abdomen. We used RT-PCR targeting nucleocapsid phosphoprotein of the COVID-19 virus. Coccolini et al. (based in Italy) noted that their RT-PCR testing also detects RNA-dependent RNA polymerase (RdRP), nucleoprotein (N), and envelope (E) [6]. Ngaserin et al. (based in Singapore) have used probe-based real-time PCR targeting nucleic acid sequences encoding RdRP and nucleocapsid phosphoprotein [8]. Hence, the variation of testing can play a confounding factor in the detection of COVID-19. High false negative rates, of up to 20% or more, of these tests are important to note [9]. Lastly, peritoneal fluid, especially in the presence of microscopic or gross perforation, can be contaminated with feces. This may be a confounding factor in virus detection in peritoneal fluid as some studies showed viral shedding in feces; however, in our case of perforated appendicitis, we still did not detect COVID-19 [4].

Current stringent guidelines on minimally invasive surgery due to concern for aerosolization of peritoneal fluids may not be necessary due to lack of convincing data of COVID-19 detection in peritoneal fluid. Of note, in the studies mentioned above, patients were tested and found positive for COVID-19 prior to the operation and hospital staff were appropriately equipped with PPE and followed current guidelines in operating room management for COVID-positive patients. Further published data are still needed on the presence of COVID-19 in peritoneal fluid to create evidence-based protocols. Until then judicious precautions should still be taken for patients with any suspicion for COVID-19 to protect frontline healthcare workers.

## Conclusions

In this case study, COVID-19 was not detected in peritoneal fluid in a patient with positive nasopharyngeal swab. Although this result seems promising that aerosolization of COVID-19 through laparoscopy, even in a perforated bowel surgery, is unlikely; further data are still needed. This study provides insight into determining current operative guidelines surrounding COVID-positive patients. Moreover, guidelines should regularly be updated as further data identify the clear pathogenicity of COVID-19.
